# Estimation of disease burden and multi-dimensional risk factors of chronic respiratory diseases among the residents near sandstone quarries in Jodhpur

**DOI:** 10.3389/fpubh.2025.1657608

**Published:** 2026-01-12

**Authors:** Suresh Yadav, Pranali A. Ahir, Dinesh S. Bhati, Rajnish Gupta

**Affiliations:** ICMR-National Institute for Implementation Research on Non-Communicable Diseases, Jodhpur, India

**Keywords:** chronic respiratory diseases (CRDs), ambient air pollution, household air pollution, sandstone mining, Rajasthan

## Abstract

**Background:**

Air pollution at mining sites is a global phenomenon; residents living around the sandstone quarries are at risk of CRDs. This is an initial research study to analyze the prevalence of CRDs, and the association of household environmental exposures, individual behaviors, socioeconomic status, and air pollution emissions from opencast sandstone quarry within 500 meters of the Soor Sagar region in Rajasthan, related to CRDs.

**Methods:**

This study represents data collected from the Soor Sagar Jodhpur area as part of a multicentric cross-sectional observational study across nine sites in India. Mapping was done using ArcGIS Pro 2.9, and participants were selected using a grid-based method. The sandstone quarry area location was mainly considered for Rajasthan, with a target of 1,000 study participant. Data were collected from pre-tested, structured questionnaire through the Kobo Toolbox and analyzed using IBM SPSS v28.0. Multivariate logistic regression and chi-square tests were applied to determine the crude and odds ratio (OR).

**Results:**

Out of 894 participants, CRDs prevalence was 12.5% (*n* = 112; occupational lung disease 8.7%, asthma 1.8%, COPD 0.6%, and tuberculosis 5.1%). NCD 6.4% (*n* = 57), (HTN & DM were prevalent). Sandstone Mine workers (OR 6.65, 95% CI: 3.06–14.45, *p* < 0.001), unemployed/former miners (OR 5.59, 95% CI: 2.32–13.47, *p* < 0.001) had increased CRDs risks. The odds of CRDs were lower for those aged 18–36 yrs. (OR 0.18, 95% CI: 0.11–0.32, *p* < 0.001), and higher for men aged 37–54 yrs. (OR 3.10, 95% CI: 2.00–4.79, *p* < 0.001). The risk of CRDs increased with illiteracy. (OR 7.41, 95% CI: 1.78–30.92, *p* = 0.006). Dust exposure was linked to an increased risk of CRDs (OR 9.00, 95% CI: 1.07–76.02, *p* = 0.044) and improper method of waste disposal (OR 4.55, 95% CI: 1.39–14.86, *p* = 0.012). The protective factor was LPG use (OR 0.29, 95% CI: 0.15–1.58, *p* < 0.01) for daily cooking (OR 0.45, 95% CI: 0.28–0.72, *p* < 0.001).

**Conclusion:**

This study highlight the prevalence of (CRDs) in this unstudied demographic of the Soor Sagar sandstone quarry area of Jodhpur. This elevated risk is strongly linked to air pollution emissions from sandstone quarries, particularly affecting mine workers and those living within 500 meters of the quarry. Air pollution from mining, combined with socioeconomic factors, personal habits, and household exposures, might increase the population’s risk of developing CRDs.

## Introduction

Chronic Respiratory Diseases (CRDs) mortality has significantly increased (46%) in India, with 94.2 deaths per 100,000 people. In contrast, the global rate slightly declined during the same period (1990–2021) (1). According to GBD (2017), India has the highest burden of CRDs, 15.69% of the Global burden (2). Estimated deaths due to Acute lower respiratory infections: 205,557; COPD: 126,708; lung cancer: 16,847 (3). These figures highlight the significant respiratory disease burdens globally and within India, primarily due to ambient air pollution (4). The second most important risk factor for premature mortality, air pollution causes about one in eight deaths worldwide and was a contributing factor in 8.1 million deaths in 2021 (5). Around 2.4 billion and 7 million people were exposed to household and combined ambient and household air pollution, with 3.2 million people dying prematurely from household air pollution, sand and dust storms contributing to increasing PM matter (6). Air pollution, especially PM_2.5_ from indoor and ambient sources, leads to 9% of CRDs in India (7). Rajasthan state is located in northwestern India, with an area of 342,239 sq. kms, contributing about 11% of India’s geographical area. It has 33 districts and with a approx. Population of 7.23 crore. Mining activities occupy around 0.54% of Rajasthan’s land. Rich in minerals, Rajasthan produces 22 major and 36 minor minerals, with 58 minerals under extraction. It is India’s sole source of lead, zinc, wollastonite, selenite, calcite, and gypsum. In Rajasthan, 137 major mineral mine leases and 16,842 minor mineral leases have been alloted; Jodhpur has one major mineral mine lease and 613 minor. However, out of 17,970 quarry licenses statewide, 13,346 are in Jodhpur, highlighting its mining importance. Mines employ 3.5 million workers, rising to 5 million by 2029–30. The district’s forts, palaces, medieval temples, structures, and local residences show its 500-year-old sandstone mining history. Sandstone from Jodhpur, known for its pink-to-gray hue, is used as masonry stone, slabs, and aslets. Key mining areas include Mandore, Soorsagar, Keru, Balesar, and Osiyan. In addition to sandstone, other rocks like rhyolite, granite, limestone, and marble are also found in Jodhpur. Local growth and jobs depend on the mining sector (8). With increasing urbanization (26.33% in 2021, projected 27.74% in 2031) (9) and a 125% vehicle growth (2010–2020) in Rajasthan are predominantly petrol/diesel vehicles (10), contributing significantly to air pollution (RAPCC, 2014). Residential biomass use (75% in rural areas) is a major source of indoor pollution (11). The Central Pollution Control Board (CPCB) of India monitors air quality via the National Air Quality Monitoring Program (NAMP), including 45 of its 966 monitoring stations based in Rajasthan (12). The National Clean Air Program (NCAP), launched in 2019, aims for a 20–30% reduction in particulate matter by 2024 in non-attainment cities (13, 14). National Ambient Air Quality Standards (NAAQS) define limits for 12 pollutants (15). The Rajasthan State Pollution Control Board implements these guidelines. Simultaneously, NCAP programs in places like as Kota and Udaipur demonstrate a decrease in NO_2_ and PM_10_ levels (16). Alwar, Jaipur, Jodhpur, Kota, and Udaipur have poor air quality. In Kota, high PM_2.5_ and PM_10_ levels (52.63 and 106.86 μg/m^3^) are linked to COPD, lung cancer, and respiratory illnesses (17). In Jodhpur, a significant portion of sandstone quarry workers suffer from silicosis (37.3%) and silico-tuberculosis (7.4%), with abnormal spirometry results in 89.2% of workers, indicating severe respiratory impairment (18). Silicosis is particularly prevalent in stone carving and sandstone quarry workers, with significant differences in occurrence and mortality rates between these sectors (19). The mining activities contribute to elevated particulate matter levels, exacerbating respiratory health issues. For instance, in Kota, Rajasthan, PM_2.5_ and PM_10_ concentrations significantly exceed national standards, contributing to increased mortality and morbidity from respiratory diseases (17). Ex-miners in Karauli reported respiratory problems such coughing up blood, shortness of breath, and wheezing as the main reason they quit their jobs (20). The situation is exacerbated by poor awareness and inadequate use of protective measures, as seen in Jodhpur, where only 8% of workers regularly wear masks (21). In Udaipur, the prevalence of tuberculosis among silicosis patients is alarmingly high at 44%, underscoring the compounded risk of respiratory infections in these environments (22). Additionally, stone carvers in Rajasthan face similar risks, with smoking and resistance to wearing masks contributing to respiratory issues (23). This study fills the knowledge gap by estimating CRDs prevalence and risk factors in the population residing in the periphery of sandstone quarry areas of Jodhpur, Rajasthan. The primary aim was to estimate the CRDs burden and explore the relative and attributable risk factors associated with CRDs, using aerosol monitors (TSI) for real-time monitoring of PM exposure. This cross-sectional study addresses the gap in disease burden and risk factors of CRDs in the population residing in the periphery of the sandstone quarry area of Soor Sagar in Jodhpur, which has not been studied earlier. The objective was to explore relative and attributable risks, providing critical data for policy and public health interventions for the large Rajasthan mining regions.

## Methodology

This data set analysis was carried out under a multicentre, cross-sectional, observational study, which incorporated a prospective, nested case-cohort study. ICMR-NIIRNCD, Jodhpur’s Institutional Ethics Committee approved the study (IEC-NIIRNCD-2022/2). The study estimates the burden of CRDs across nine sites in India, representing diverse air quality and socio-economic settings. Jodhpur’s mining areas were included at the Rajasthan site only alongside urban/rural comparisons. The other 9 study sites of ICMR and medical college. This secondary analysis evaluates CRDs prevalence and risk factors in areas near sandstone quarries, considering high PM exposure year-round except during the rainy season. Further, Jodhpur is the gateway to the Great Indian Thar Desert and was exposed to amorphous silica dust through occasional sand storms, thus leading to PM exposure in the population.

### Site descriptions

**Figure 1:**
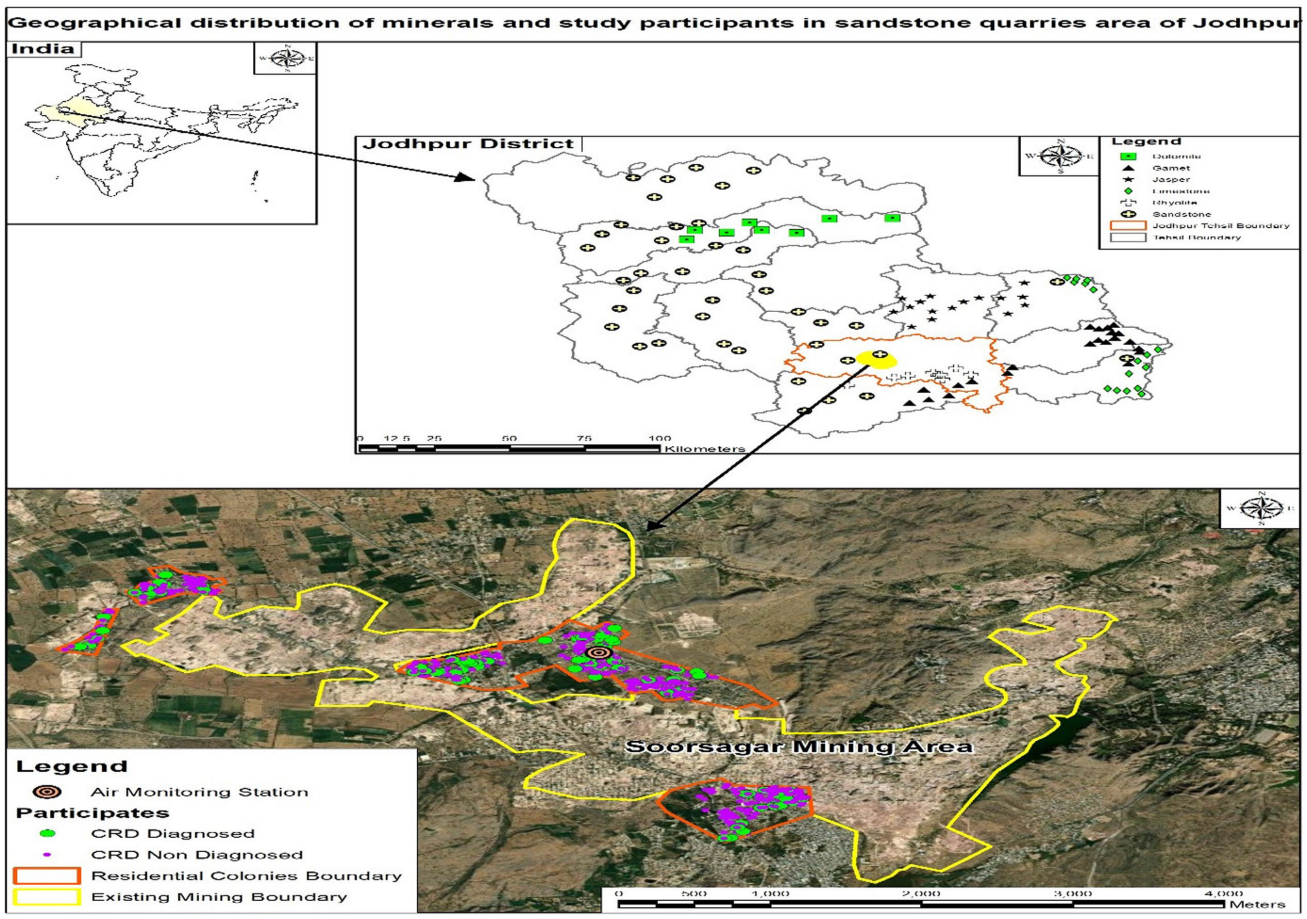
Geographical distribution of minerals and study participants in sandstone quarry area of Jodhpur.

In Rajasthan, Jodhpur is located on the eastern edge of the Thar Desert. The main industry in the Jodhpur district is sandstone quarrying, mostly located 8–10 km north of Jodhpur, including Mandore, Fedusar, Soorsagar, Kali Beri, and Keru sandstone quarry regions are located 8–10 km north of Jodhpur (24) (Figure 1). Groundwater analysis done in this mining prone area indicates raised concentrations of fluoride and nitrate in several regions; some samples also show increased alkalinity and chloride, but other parameters remain within acceptable ranges (25). The landscape around Soorsagar is rugged with little extensive agriculture; yet, at the northern periphery beyond the rocky elevations, some seasonal Kharif and Rabi crops are cultivated. Pearl millet, moong, moth beans, jowar, cluster beans, are the main Kharif crops grown in the region. Ambient monitoring at three locations around the quarry sites documented both peak and off-peak hours. The estimation of particulate matter (PM) exposure included quarry proximity, duration of site presence, occupational exposure, and other pertinent criteria. The monitoring occurred in October (winter); during this season, the wind originates from the northeast at low velocities of around 3–4 km/h, but in summer, it emanates from the southwest at greater speeds of about 9–12 km/h (26).

### Sample size calculation

Using GBD 2016 statistics (COPD: 4.2%, Asthma: 2.9%), the research sample size was estimated. The mining region site was only considered for Rajasthan, and purposeful sampling ensured variable air quality exposure each site.


$$n=(z^{2*}p^{*}(1–p))/d^{2}$$

*Z* = 1.96 (95% confidence level).

*p* = estimated prevalence of disease (e.g., asthma = 0.029 or COPD = 0.042).

*d* = margin of error (e.g., 0.015 or 1.5%).

Asthma (2.9%), 1.5% precision:


$$n=\frac{{\left(1.96\right)}^{2}.0.029.(1-0.029)}{{\left(0.015\right)}^{2}}\approx 480$$

COPD (4.2%), 1.5% precision:


n≈705

The overall response rate was 89.4% (894 of 1,000 targeted participants). The remaining 10.6% non-response was due to inaccessibility or unavailability of household members during the data collection period.

### Study participant enrolments

The participants in the study were enrolled from the colonies of Soor Sagar (Kaliberi, 250 m from the mine, Ambedkar Nagar, 500 m from the mine, Sodo ki Dhani, 250 m from the mine, Utho ki Dhani, 270 m from the mine) which are within a 500 m distance range from the sandstone quarry area. After identifying the colonies, a GIS-based map was developed, and a grid sampling method was applied to enroll participants. To map residential settlements near the sandstone quarry. In this area, we created a polygon of residential settlements based on the mining area. We created a grid of 250 × 250 m according to the residential settlement boundary.compared to a larger grid and to enable more realistic local burden estimates for populations residing at the periphery of quarries. Furthermore, the habitations are also scattered in pockets due to the rocky terrain. We utilized the Existing Land Use Plan map from the local authority, Jodhpur Development Authority, JoDA (27) for georeferencing the Existing Land Use Plan using existing landmark points. Participants aged 18–75 years and residing for≥5 years in study areas were included. Those with cognitive impairment, congenital lung diseases, or active infections were excluded. CRDs patients were enrolled for longitudinal assessment.

This study targeted 1,000 mining-area participants. However, only 894 met the inclusion criteria (age range, availability, and willingness). Many households were inaccessible during data collection. A field investigator visited the selected households, explained the study, and obtained written consent from each respondent. In case of refusal, the immediate neighbor household was approached till a willing household was found in the specified grid. Only one eligible member was selected from each household during this study using the Kish sampling method.

### Data collection

In this mining area, the data collection was carried out in October 2024. The research team visited the area for data collection early in the morning. Ambient air monitoring was conducted for PM₁, PM₂.₅, and PM₁₀ over 3 days, with 8 h of monitoring each day, using the TSI DustTrak DRX (EP) aerosol monitor. Data was collected using the Kobo Toolbox using a validated, pretested, structured questionnaire to collect vital data like Location Details, GPS co-ordinates of surveyed houses, Demographic profile of the participants, Household and Environmental Factors, Lifestyle assessing waste handling, cooking fuel, Occupational history, CRDs diagnosis. CRDs diagnosis was established by affirmative record prescription of previous diagnosis of CRDs and past history of intake medication in the past 12 months, which was confirmed by the Medical Officer.

### Statistical methods

In this study, the primary dependent and outcome variable was the diagnosis of (CRDs), classified as a binary variable (diagnosed/undiagnosed) for population-level assessments to simplify the analysis, enabling assessment of associations with risk factors, while maintaining clinical relevance through physician-confirmed diagnoses. Additional outcomes included the presence of Pulmonary tuberculosis (PTB) the independent and exposure variables included air pollutant exposure, household fuel type, cooking frequency, ventilation type, and dust presence in the household. Predictors are Demographic (age, gender, education), and type of work, while potential confounders include household waste disposal methods and kitchen ventilation. Age and gender were considered effect modifiers for stratified analyses. All quantitative variables were presented as categorical variables grouped into categories based on distribution and public health relevance. Grouping allowed for meaningful comparisons in logistic regression models.

We used IBM SPSS version 28.0 for all of our analyses. The frequencies and percentages were presented using descriptive statistics. The relationship between CRDs and independent factors was examined using chi-square tests in bivariate analysis. Odds ratios (ORs) with 95% confidence intervals (CIs) for possible risk factors were calculated using multivariate logistic regression. In order to evaluate risks across time, we computed relative risk and attribution risk. Age and gender were used as criteria for subgroup analysis. Using stratified models, we evaluated how different exposures (such fuel type and ventilation) interacted with one another. Minimal data was missing; individuals whose answers lacking were not included in the corresponding analyses. When comparing findings with complete-case analysis, sensitivity studies made sure to avoid extreme values. All of the studies were conducted with the aim of adequately evaluating the risk factors linked to CRDs by identifying significant associations and controlling for any confounding.

## Results

The data was analyzed and tabulated in frequency and percentage distribution in Table 1 to interpret the results. The study findings showed that CRDs was high among the 894 participants residing near the sandstone quarry area. 12.5% (*n* = 112/894) were diagnosed with CRDs, including both obstructive and restrictive respiratory diseases, and tuberculosis prevalence was 5.1% (*n* = 46/894); the prevalence was determined via prescription and clinical documentation. The Occupational Lung Diseases was the most frequent (8.7%, *n* = 78/894), followed by asthma (1.8%, *n* = 16/894) and COPD (0.6%, *n* = 5/894). Combined CRDs diagnoses were rare (<1% each). Non-communicable chronic diseases were reported in 6.4% (*n* = 57/894), with hypertension (2.8%, *n* = 25/894) and diabetes (0.4%, *n* = 4/894) being the most common. Comorbidities like diabetes with hypertension (1.1%, *n* = 10/894) and hypertension with psychiatric illness (0.2%, *n* = 2/894) were infrequent.

**Table 1:** Frequency and percentage of socio-demographic, environmental, and behavioral factors, health status and outcome variables.

Section-1	Socio-economic and demographic characteristics	Category	Frequency(n)	Percentage (%)
1.	Age	18–36	480	53.7
		37–54	275	30.8
		55–75	139	15.5
2.	Gender	Male	439	49.1
		Female	455	50.9
3.	Education	Illiterate	395	44.2
		Up to the 5^th^ class	234	26.2
		Secondary Pass	188	21.0
		Graduation and above	77	8.6
4.	Type of work	Mine worker	202	22.6
		House wife	351	39.3
		Unemployed	75	8.4
		Agriculture	12	1.3
		Industry	25	2.8
		Construction	45	5.0
		Students	53	5.9
		Self-employed	131	14.7
Section-2	Household and environmental exposure factors	Category	Frequency(n)	Percentage (%)
1.	Fuel used for cooking in the house	Coal	1	0.1
		Wood/dung cake	67	7.5
		Kerosine oil	1	0.1
		LPG, PNG	478	53.5
		LPG, PNG, coal	3	0.3
		LPG, PNG, coal, wood, dung cake	1	0.1
		LPG, PNG, wood dung cake, electricity	336	37.6
		LPG, PNG, wood dung cake, electricity	1	0.1
		Coal, wood, dung cake	6	0.7
2.	Household wastage disposable system	Garbage collection van	88	9.8
		Garbage collection van, throw in the dumping yard, throw on road	7	0.8
		Throw in the dumping yard	135	15.1
		Garbage collection van, throw on road	14	1.6
		Garbage collection van, throw in the dumping yard	137	15.3
		Throw in the dumping yard, throw on the road	419	46.9
		Throw on the road	71	2.9
		Other	23	2.6
3.	Kitchen, separate living room	No	249	27.9
		Yes	645	72.1
4.	Source ventilation kitchen	Chimney	302	33.8
		Exhaust	55	6.2
		Vent	436	48.8
		Extra hood system	3	0.3
		No ventilation system	98	11.0
5.	Frequency cooking	Daily	338	37.8
		Occasionally	28	3.1
		Once a week	19	2.1
		Not applicable	509	56.9
6.	Work environmental exposure	Pollutants (Gaseous)	2	0.2
		Dust	248	27.7
		Heat	49	5.5
		Volatile organic compounds (VOC)	2	0.2
		Pollutants, dust	37	4.1
		Pollutants, dust, heat	32	3.6
		Pollutants, heat	1	0.1
		Dust, volatile organic compounds (VOC)	3	0.3
		Dust, heat	54	6.0
		No response	466	52.1
7.	Work environmental exposure	Exposed	428	47.9
		Not exposed	466	52.1
Section-3.	Tobacco smoke factors	Category	Frequency(n)	Percentage (%)
1.	Present smoking practice	Daily	41	4.6
		Occasionally	18	2.0
		Never smoked	835	93.4
Section-4.	Health status and outcome variables	Category	Frequency(n)	Percentage (%)
1.	Tuberculosis	Yes	46	5.1
		No	848	94.9
2.	CRDs	Diagnosed	112	12.5
		Not diagnosed	782	87.5
3.	CRDs diagnosed	Asthma	16	1.8
		COPD	5	0.6
		Occupational lung diseases	78	8.7
		Pneumonia	4	0.4
		Asthma, COPD, occupational lung diseases	2	0.2
		COPD, occupational lung diseases	5	0.6
		COPD, pneumonia	1	0.1
		Asthma, occupational lung diseases	1	0.1
		Not diagnosed	782	87.5
4.	Non-communicable chronic diseases	Diabetes	4	0.4
		Hypertension	25	2.8
		Coronary artery disease	2	0.2
		CKD	1	0.1
		Liver cirrhosis	1	0.1
		Any psychiatric illness	8	0.9
		Diabetes, hypertension	10	1.1
		Diabetes, coronary artery disease	1	0.1
		Diabetes, CKD	1	0.1
		Hypertension, coronary artery disease	1	0.1
		Hypertension, congestive heart failure	1	0.1
		Hypertension, any psychiatric illness	2	0.2
		No respiratory disease	837	93.6

Socio-economic and Demographic Characteristics showed that the majority of participants (53.7%, *n* = 480/894) were aged 18–36 years, followed by 37–54 years (30.8%, *n* = 275/894) and 55–75 years (15.5%, *n* = 139/894). Gender distribution was nearly equal (male: 49.1%, *n* = 439/894; female: 50.9%, *n* = 455/894). Most participants were illiterate (44.2%, *n* = 395/894) or had primary education (26.2%, *n* = 234/894). Only 8.6% (*n* = 77/894) had a graduation or higher degree. Housewives (39.3%, *n* = 351/894) and mine workers (22.6%, *n* = 202/894) were the most common occupational groups.

Household and Environmental Exposure Factors indicated that the primary cooking fuel was LPG/PNG (53.5%, *n* = 478/894), while 37.6% (*n* = 336/894) used a combination of LPG, wood, dung cake, and electricity. 46.9% (*n* = 419/894) of participants disposed household waste by throwing it in dumping yards or on roads. 72.1% (*n* = 645/894) had a separate kitchen, and 85.8% (*n* = 767/894) reported cross ventilation. 48.8% (*n* = 436/894) relied on vents for kitchen ventilation, while 11.0% (*n* = 98/894) had no ventilation system. 56.9% (*n* = 509/894) reported never cooking, while 37.8% (*n* = 338/894) cooked daily. 47.9% (*n* = 428/894) were exposed to Work Environmental exposure, with dust being the most common (27.7%, *n* = 248/894) 52.1% (*n* = 466/894) reported no exposure.

Tobacco Smoke Exposure Factors found that the majority of participants (93.4%, *n* = 835/894) had never smoked, while a small proportion reported daily (4.6%, *n* = 41/894) or occasional (2.0%, *n* = 18/894) smoking. The analysis excluded no participants due to missing data.

The associations between socio-demographic, environmental, and behavioral factors with (CRDs) in the population residing near Jodhpur’s Soor Sagar sandstone quarry areas (Table 2) indicate that 112/894 participants (12.5%) were diagnosed with (CRDs). The participants diagnosed with (CRDs) are based on clinical records and prescriptions, which include both obstructive and restrictive types. Thus, only consistently available medical records and prescription variables were analyzed for CRDs-related outcomes.

**Table 2:** Associations between socio-demographic, environmental, and behavioral factors with chronic respiratory disease (CRDs) in the population residing near sandstone quarry areas of Soor Sagar, Jodhpur.

Variable	Diagnosed with CRDs (*n* = 112)	Undiagnosed (*n* = 782)	OR (95% CI)	*p*-value	Chi-square
Associations between age and chronic respiratory disease (CRDs)					
18–36	26 (23.2%)	454 (58.1%)	0.184 (0.106–0.321)	<0.001	χ^2^: 49.515, df = 2, *p* < 0.001
37–54	53 (47.3%)	222 (28.4%)	0.767 (0.469–1.255)	0.291	
55–75	33 (29.5%)	106 (13.6%)	Reference category	-	
Associations between gender and chronic respiratory disease (CRDs)					
Male	81 (72.3%)	358 (45.8%)	3.095 (1.999–4.792)	<0.001	χ^2^: 27.614, df = 1, *p* < 0.001
Female (ref.)	31 (27.7%)	424 (54.2%)	1.000 (ref.)	-	
Associations between education level and chronic respiratory disease (CRDs)					
Illiterate	67 (59.8%)	339 (43.4%)	7. 412(1.776–30.924)	0.006	χ^2^: 15.586, df = 3, *p* < 0.001
Up to 5^th^ class	27 (27.9%)	196 (25.1%)	5.166 (1.199–22.261)	0.028	
Secondary pass	16 (14.3%)	172 (22.0%)	3.488 (0.782–15.552)	0.101	
Graduation & above	2 (1.8%)	75 (9.6%)	Reference category	-	
Association of type of work and chronic respiratory disease (CRDs)					
Mine worker	61 (54.5%)	141 (18.0%)	6.652 (3.062–14.447)	<0.001	χ^2^: 106.935, df = 7, *p* < 0.001
Housewife	23 (20.5%)	328 (41.9%)	1.078 (0.470–2.474)	0.859	
Unemployed	20 (17.9%)	55 (7.0%)	5.591 (2.320–13.471)	<0.001	
Self-employed	8 (7.1%)	123(15.7%)	Reference Category	-	
Agriculture-related work	0 (0.0%)	12 (1.5%)	Not estimable due to zero frequencies	-	
Industrial-related work	0 (0.0%)	25 (3.2%)	Not estimable due to zero frequencies	-	
Construction-related work	0 (0.0%)	45 (5.8%)	Not estimable due to zero frequencies	-	
Student	0 (0.0%)	53 (6.8%)	Not estimable due to zero frequencies	-	
Association of type of work and pulmonary tuberculosis (PTB)					
Mine Worker	22 (10.9%)	180 (89.1%)	3.881 (1.306–11.534)	0.015	χ^2^: 24.507, df = 7, *p* < 0.001
Housewife	13 (3.7%)	338 (96.3%)	1.221 (0.391–3.815)	0.731	
Unemployed/ former miner	6 (8%)	69 (92.0%)	2.761 (0.735–10.117)	0.125	
Agriculture-related work	1 (8.3%)	11 (91.7%)	2.886 (0.296–28.113)	0.361	
Self-employed	4 (3.1%)	127(96.9%)	Reference Category	-	
Industrial work	0 (0.0%)	25 (100%)	Not estimable due to zero frequencies	-	
Construction	0 (0.0%)	45 (100%)	Not estimable due to zero frequencies	-	
Student	0 (0.0%)	53 (100%)	Not estimable due to zero frequencies	-	
Association of work environmental exposure * and chronic respiratory disease (CRDs)					
Exposed to work environmental exposure	54 (48.2%)	87(11.1%)	7.438(4.826–11.462)	<0.001	χ^2^: 101.44, df = 1, *p* < 0.001
Not exposed to work: environmental exposure	58(51.8%)	695 (88.9%)	Reference Category	-	
Association of household fuel use for cooking and chronic respiratory disease (CRDs)					
Coal, wood, dung cake	2 (1.8%)	4 (0.5%)	1.59 (0.267–9.525)	0.609	χ^2^: 29.059, df = 8, *p* < 0.001
LPG PNG	40 (35.7%)	438 (56%)	0.291 (0.152–1.577)	<0.001	
LPG PNG coal	1 (0.9%)	2 (0.3%)	1.594 (0.135–18.753)	0.711	
LPG PNG wood dung cake	52 (46.4%)	284 (36.3%)	0.584 (0.309–1.101)	0.584	
LPG, PNG, wood, dung cake, electricity	1 (0.1%)	0 (0%)	3.064E-7 (000–0)	-	
Wood / dung cake	16 (14.3%)	51 (6.5%)	Reference		
Coal	0 (0%)	1 (0.1%)	Not estimable due to zero frequencies	0.996	
Kerosene oil	0 (0%)	1 (0.1%)	Not estimable due to zero frequencies	-	
LPG, PNG, coal, wood, dung cake	0 (0%)	1 (0.1%)	Not estimable due to zero frequencies	-	
Association of household waste disposal systems and chronic respiratory disease (CRDs)					
Throw in the dumping yard, throw on road	70 (62.5%)	349 (44.6%)	4.546 (1.391–14.861)	0.012	χ^2^ = 27.188, df = 7, *p* < 0.001
Garbage collection van & trash dumping yard	24 (21.4%)	113 (14.5%)	4.814 (1.397–16.592)	0.013	
Garbage collection van & throw on road	2 (1.8%)	12 (1.5%)	3.778 (0.570–25.276)	0.168	
Garbage collection van	4 (3.6%)	84 (10.7%)	1.079 (0.234–4.988)	0.922	
Other method	1 (0.9%)	22 (2.8%)	1.030 (0.102–10.814)	0.980	
Throw in the dumping yard	8 (7.1%)	127 (16.2%)	1.428 (0.367–4.59)	0.608	
Throw on the road	3(2.7%)	68(8.7%)	Reference	-	
Garbage collection van throw in the dumping yard throw on the road	0 (0.0%)	7 (0.9%)	Not estimable due to zero frequencies	-	
Association of the presence of dust in the house and chronic respiratory disease (CRDs)					
A thick layer of dust	9 (8%)	27 (3.5%)	9.000 (1.066–76.017)	0.044	χ^2^: 19.539, df = 3, *p* = 0.003
On wiping the surface	24 (21.4%)	133 (17%)	4.872(0.632–37.572)	0.129	
On wiping the surface, a thin layer of dust is visible	19 (17%)	75 (9.6%)	6.840 (0.873–53.582)	0.067	
On wiping the surface, a thin layer of dust and a thick layer of dust were visible.	1 (0.5%)	7 (0.9%)	3.857 (0.214–69.666)	0.361	
A thin layer of dust is visible	51 (45.5%)	485 (62%)	2.839 (0.378–21.332)	0.311	
A thin layer of dust is visible, a thick layer of dust	7 (6.3%)	28 (3.6%)	6.750 (0.778–58.586)	0.083	
No response	1 (0.5%)	27 (3.5%)	Reference	-	
Association of frequency of cooking and chronic respiratory disease (CRDs)					
Daily	26 (23.2%)	312 (39.9%)	0.454 (0.284–0.723)	0.001	χ^2^: 12.716, df = 3, *p* = 0.005
Occasionally	3 (2.7%)	25 (3.2%)	0.653 (0.193–2.215)	0.494	
Once a week	4 (3.6%)	15 (1.9%)	1.451 (0.469–4.488)	0.518	
Not applicable (N/A*)	79 (70.5%)	430 (55.0%)	Reference	-	
Association of smoke exposure factors and chronic respiratory disease (CRDs)					
Daily An adult who has smoked at least 100 cigarettes in their lifetime, and also now smokes every day	9 (8.0%)	32 (4.1%)	1.101 (0.249–4.858)	0.899	χ^2^: 3.498 df = 2, *p* = 0.174
Occasionally An adult who has smoked at least 100 cigarettes in their lifetime, who smokes now, but does not smoke every day	2 (1.8%)	16 (2.0%)	2.250 (0.434–11.663)	0.334	
Never smoked	101 (90.2%)	734 (93.9%)	(Reference)	-	
Association of kitchen ventilation and chronic respiratory disease (CRDs)					
Vent (Any opening in the kitchen)	61 (54.5%)	375 (48.0%)	1.064 (0.559–2.024)	0.851	χ^2^: 4.084, df = 4, *p* = 0.395
Chimney	35 (31.3%)	267 (34.1%)	0.857 (0.433–1.695)	0.658	
Exhaust fan vent	3 (2.7%)	52 (6.6%)	0.377 (0.103–1.387)	0.142	
No ventilation system	13 (11.6%)	85 (10.9%)	Reference	-	
Extra hood system	0 (0.0%)	3 (0.4%)	Not estimable due to zero frequencies	-	

*Workplace environmental exposures include airborne gaseous pollutants (criteria and hazardous, e.g., VOCs), inhalable particulate matter (dust), and thermal stress (heat). Dust exposure refers to the inhalation of airborne dust particles, which can have harmful health effects, including respiratory issues and various forms of lung diseases such as fibrosis. It encompasses both total inhalable dust and respirable dust, with exposure limits set to minimize health risks (45). *Cooking not applicable cases were primarily observed in older adults, ill individuals, or interviewed men.

CRDs was associated with the type of work using multi-nominal logistic regression analysis, which suggests that among diagnosed *n* = 112, 61 (54.5%) mine workers suffered from CRDs. In comparison, 141/782 (18.0%) were not diagnosed with CRDs, with an odds ratio (OR) of 6.652 (95% CI: 3.062–14.447, *p* < 0.001). Unemployed or former miners had an OR of 5.591 (95% CI, 2.320–13.471, *p* < 0.001). Other occupations, including homemakers and those involved in agriculture, construction, or industrial work, did not significantly correlate with CRDs (*p* = 0.05). Chi-square tests confirmed significant differences in CRDs diagnosis across types of work (χ^2^: 106.935, *p* < 0.001).

The association of Pulmonary tuberculosis (PTB) with the type of work using multi-nominal logistic regression analysis suggest that the sandstone quarry workers had a higher tuberculosis prevalence, n = 22/112 (10.9%; OR) of 3.881 (95% CI: 1.306–11.534, *p* = 0.015). Other occupations, including homemakers and those involved in agriculture, construction, or industrial work, did not significantly correlate with Pulmonary tuberculosis (PTB). The chi-square test indicated a significant association (χ^2^ = 24.507, df = 7, *p* < 0.001).

The Association of Demographic Factors with CRDs revealed that among diagnosed participants, *n* = 112 were 18-36 yrs. Age group *n* = 26/112 (23.2%), OR: 0.184 (95% CI: 0.106–0.321; *p* < 0.001) and 37–54 yrs. age group show lower odds supported by chi-square χ^2^: 49.515 (df = 2, *p* < 0.001) overall association was statistically significant across all categories. A total of 81 out of 122 males (72.3%) and 31 out of 122 female participants (27.7%) were found to have CRDs, with an odds ratio (OR) of 3.095 (95% CI: 1.999–4.792), *p* < 0.001. This finding was verified by the chi-square test χ^2^: 27.614 (df = 1, *p* < 0.001). A greater incidence of CRDs was seen in illiterate persons (*n* = 67/112; 59.8%; OR: 7.412, 95% CI: 1.776–30.924, *p* = 0.006), but no significant relationships were found in the categories of up to 5 classes, secondary pass, and graduate. A chi-square test (χ^2^ = 15.586, df = 3, *p* < 0.001) validated the correlation between decreased levels of education and elevated risk of CRDs.

The association between environmental factors and CRDs signifies that exposure to Work Environmental exposure n = 54/112 (48.2%) significantly increased CRDs risk (OR: 7.438, 95% CI: 4.826–11.462, *p* < 0.001), Chi-square analysis (χ^2^ = 101.44, df = 1, *p* < 0.001).

Similarly, 26 out of 112 (23.2%) cook at least once a day. (odds ratio: 0.454, 95% confidence interval: 0.284–0.723, *p* < 0.001) The chi-square test (χ^2^ = 12.716, df = 3, *p* = 0.005) and cooking using LPG, PNG, wood, and dung cake (OR: 0.291, 95% CI: 0.152–1.577, *p* < 0.001). In comparison to the reference group, a decreased likelihood of CRDs was linked to chi-square analysis (χ^2^: 29.059, df = 8, *p* < 0.001).

**Figure 2:**
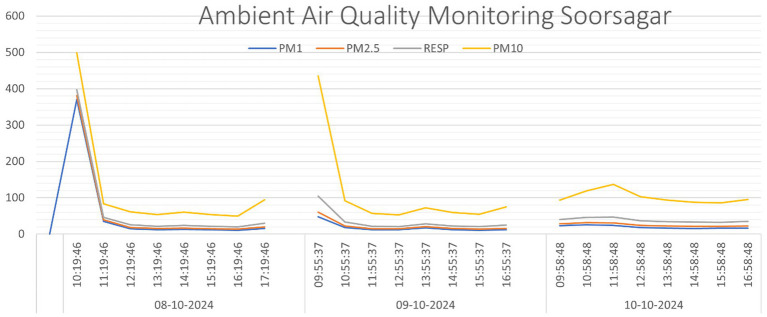
presents hourly average concentrations of PM_1.0_, PM_2.5_, PM_RES_, and PM_10_, showing higher PM levels during the first 1 to 2 h of each monitoring session. A slight increase in PM concentrations was observed around 2 O′ Clock and in the evening. Notably, the early spikes in PM levels on the first 2 days are likely attributed to nearby construction activities. The three-day average concentrations were: PM_1.0_: 33 μg/m^3^, PM_2.5_: 37 μg/m^3^, PM_RES_: 48 μg/m^3^, and PM_10_: 111 μg/m^3^. The PM_10_ levels exceeded the prescribed air quality standard, likely due to nearby mining operations. For comparison, the Rajasthan State Pollution Control Board (RSPCB) reported an annual PM_10_ average of 123.10 μg/m^3^, which aligns closely with the PM_10_ levels recorded in Soorsagar during this study (44).

Statistical analysis showed that a thick layer of dust on surfaces was substantially linked with CRDs (OR: 9.000, 95% CI: 1.066–76.017, *p* = 0.044). The chi-square analysis (χ^2^: 19.539, df = 3, *p* = 0.003) showed greater risks of CRDs. Throwing trash in the dump or on the road increases the risk of CRDs (OR: 4.546, 95% CI: 1.391–14.861, *p* = 0.012). CRDs were analyzed using Chi-square testing (χ^2^ = 27.188, df = 7, *p* < 0.001). Use of exhaust fan and chimney have lower odds with CRDs. Non-significant chi-square analysis (χ^2^: 4.084, df = 4, *p* = 0.395). Daily smokers had higher risks of CRDs (OR: 1.101, 95% CI: 0.249–4.858, *p* = 0.899), using Chi-square analysis (χ^2^: 3.498, df = 2, *p* = 0.174), although the difference is less statistically significant (Figure 2).

## Discussion

The study examined the prevalence of CRDs (among the population residing near Jodhpur’s Soor Sagar sandstone quarry areas). The study established that the prevalence of respiratory diseases was high, and *n* = 112/894 (12.5%) of the study participants were diagnosed with CRDs, in which 78 (8.7%) Occupational Lung Diseases were remarkably higher, suggesting that sandstone quarry areas may have a higher respiratory disease burden. Studies have shown that individuals living near mining sites are at high risk of CRDs and higher healthcare expenses (28). Particulate matter (PM) levels near mining operations frequently exceed national air quality guidelines, resulting in respiratory disorders among residents (29). Residents in the mining and industrial areas of Keonjhar District, Odisha, experienced respiratory difficulties, including asthma and tuberculosis, caused by air pollution from mining activities (30). In the Jodhpur district, the studies have reported higher prevalence of silicosis (37.3%) and silico-tuberculosis (7.4%) among sandstone quarry workers. The abnormal spirometry results have been reported in 89.2% and abnormal chest x-rays in 42% (18).

The significant association of risk factors, as shown in Table 2, has been assessed as a secondary objective of the study participants, who were sandstone quarry workers, exhibited remarkably higher odds of CRDs and Pulmonary tuberculosis (PTB) (OR: 3.881, 95% CI: 1.306–11.534, *p* = 0.015) compared to reference category indicating occupational exposure as a critical risk factor. During data collection, unemployed respondents stated that they were former miners, and others who resided close to mining sites had elevated odds ratio of CRDs (OR: 5.591, CI: 2.320–13.471, *p* < 0.001). These findings correlate with literature that highlights occupational hazards such dust, silica, and poor ventilation, and a lack of safety measurements are major risk factors for respiratory disease. Similarly, in the Karauli district, current and ex-mine workers reported high respiratory symptoms, with ex-miners suffering from symptoms such as coughing up blood and dyspnoea (20). Silicosis is notably high in the stone carving and sandstone quarry industries, with significant differences in occurrence and mortality rates between these sectors (19). Workers in Budhpura Village also faced severe health impacts from silicosis due to inadequate health protection and weak law enforcement (31). The results of this study are in line with what has been found in this area, where the Pneumoconiosis Board of Jodhpur District has confirmed over 3,600 cases of silicosis. This highlights the importance of taking steps to prevent it, such as controlling dust and wearing personal protective equipment (PPE) (32). Furthermore, residential colonies should not be located near mining areas to prevent the CRDs burden on the population of Jodhpur city.

The data show that younger age (18–36 years) was substantially linked to lower odds ratio (OR: 0.184, 95% CI: 0.106–0.321, *p* < 0.001) of CRDs (), χ^2^: 49.515, df = 2, *p* < 0.001 compared to older persons (55–75 years). Similarly, male (OR: 3.095, 95% CI: 1.999–4.792, *p* < 0.001), χ^2^: 27.614, df = 1, *p* < 0.001, and education level showed reliable associations, with males (OR: 3.095) and education illiterate category (OR: 7.412, 95% CI: 1.776–30.924, *p =* 0.006), having a higher risk of CRDs. The CIs for multiple variables were wide due to a smaller sample size. The results suggest that demographic risk factors could influence disease burden and that targeted respiratory health interventions should focus on geriatric, and vulnerable demographics.

Illiteracy among stone carvers makes them less likely to use masks, which worsens respiratory concerns (23). The Rajasthan government’s initiatives, such as the Pneumoconiosis & Silicosis Policy, aim to address these issues by providing screening and relief to affected individuals. However, the effectiveness of these measures is limited by the population’s literacy levels (19). Illiterate adults living near quarry mining sites are at a higher risk of CRDs due to environmental exposures and socio-economic vulnerabilities, lack of employment, necessitating targeted interventions to improve awareness of healthcare access and promote the use of personal protective measures (33, 34).

The study further demonstrates a significant association between working environmental exposure and CRDs; participants’ exposure to airborne pollutants, dust, or heat had 7.4-fold greater odds of disease (OR = 7.438; 95% CI 4.826–11.462; χ^2^ = 101.44, df = 1 *p* < 0.001). On a regular basis, cooking was found to be protective (OR = 0.454; 95% CI 0.284–0.723; *p* < 0.001; χ^2^ = 12.716, df = 3 *p* = 0.005), possibly due to the use of clean fuels (LPG/PNG) and adequate ventilation among those who cook regularly suggesting potential adaptive or ventilation factors, whereas less frequent or weekly cooking showed non-significant associations. Fuel type used analysis revealed LPG/PNG users had a lower odds ratio (OR: 0.291, CI: 0.152–1.577, *p* < 0.001 χ^2^: 29.059, df = 8, *p* < 0.001), but broad CIs were seen in some mixed-fuel users due to small sample numbers and data sparsity. Overall, our findings emphasize the necessity of lowering occupational and household air pollution as preventive measures against CRDs in sensitive populations.

The studies done in Kota reported PM_2.5_ and PM_10_ values of 52.63 and 106.86 μg/m^3^, surpassing national requirements and associated with high estimated COPD and lung cancer incidences (35, 36). Particulate matter (PM_2.5_) was connected to 12,867 natural deaths in Alwar district (37). CRDs and home air pollution were linked in a Jaipur research, which found worse lung function in rural people (38). Kerosene, biomass, cow dung cakes, coal, and LPG homes emit NO_2_, SO_2_, CO, SPM, and RSPM, contributing 6% of the National Burden of Diseases in India (39). Studies have indicated that rural households in Rajasthan still rely on biomass fuels, with 43.1% of women in Jodhpur primarily using biomass, despite the availability of cleaner alternatives like liquified petroleum gas (LPG) (40). In rural areas, biomass and coal used for cooking result in high levels of particulate matter (PM_10_ and PM_2.5_) exposure, leading to increased health risks such as asthma, stroke, ischemic heart disease, and CRDs (41). The Indian government’s initiatives, Pradhan Mantri Ujjwala Yojana (PMUY), aim to promote the adoption of cleaner fuels like LPG to mitigate these health risks (42).

In the current study, Table 2, the individuals disposing of waste via the dumping yard and roadside demonstrated higher odds of CRDs (OR = 4.546, *p* = 0.012). Kitchen and cross ventilation showed no significant associations with CRDs (*p* = 0.05). Dust presence within households showed significance (χ^2^ = 19.539, *p* = 0.003), particularly thick dust layers (OR = 9.000, *p* = 0.044). Smoke exposure was not statistically significant (*p* = 0.174). These findings suggest that environmental hygiene factors, especially waste disposal and indoor dust, significantly influence CRDs risk.

In this study, improper home waste management, particularly throwing in the dumping yard or on the road, was strongly associated with CRDs. (OR: 4.546, 95% CI: 1.391–14.861, *p* = 0.012) demonstrating significant health concerns associated with unregulated disposal procedures. Similarly, families exposed to a thick coating of dust had a higher risk (OR: 9.000, 95% CI: 1.066–76.017, *p* = 0.044), indicating the importance of indoor air quality effects in respiratory pathology. However, factors such as daily smoking (OR: 1.101, 95% CI, 0.249–4.858, *p* = 0.899) did not have statistically significant relationships from underreporting smoking status because of social desirability bias, fear of disenrollment the government’s silicosis policy. This underreporting had affected the precise effect of smoking on the risk of CRDs.

Previous PM_2.5_ and PM_10_ levels surpass national ambient air quality guidelines in Kota and Alwar, causing serious health hazards such COPD, lung cancer, and acute lower respiratory infections (36, 37). Smoking further exacerbates respiratory health issues, as evidenced by a study in Jaipur, which found that smokers exhibited significantly lower pulmonary function test parameters compared to non-smokers, with a higher prevalence of obstructive patterns (43). The risks are aggravated by occupational exposure, as experienced by sandstone quarry rs and carvers in Rajasthan, along with smoking habits and resistance to protective measures like masks, contributing to respiratory problems (23).

Smoking status was underreported, leading to misclassification and possible underestimation of its association with CRDs. Environmental exposure assessment over 3 days may not reflect long-term variability, causing exposure misclassification. Recall that bias could affect self-reported data on fuel use, pollutant exposure, and occupational history. A larger sample size could have resulted in a more precise estimation of the study variables.

### Strengths

This study provided the first community-based prevalence data for CRDs in the Soor Sagar mining area, establishing a baseline for future monitoring. Its innovative use of GIS mapping and analysis offers a model for similar studies. The large sample size and detailed assessment of exposures strengthen evidence to guide interventions.

### Limitations

This cross-sectional study has several limitations, including the inability to establish causal relationships between exposures and CRDs outcomes. Recall bias may have affected self-reported data. Assessment of individual-level PM exposure was difficult due to limited facilities. The findings were restricted to a specific sandstone quarry area, thereby limiting their generalizability.

## Conclusion

This study contributes the context specific cross-sectional survey being conducted and the study used unique techniques and methods to estimate the CRDs burden and risk variables in Jodhpur area residents living near sandstone quarries. Spatially mapping residential areas and screening participants via Grid-based sampling is a critical milestone. With exact identification of mining region residents, focused data collection and risk evaluations were achieved. In addition, real-time particulate matter (PM) monitoring using the TSI DustTrak DRX aerosol monitor is an advanced method for assessing air pollution exposure, particularly PM_10_, PM_2.5_, PM_10_, and PMRES levels, which are crucial for assessing mining risk health risks. Innovatively, the research utilizes multivariate logistic regression and chi-square testing to examine the relationship between socio-economic, environmental, and behavioral variables and CRDs. Collectively, these statistical methods and extensive exposure evaluations give a complete picture of population hazards. Because it addresses a significant gap in data on the health implications of sandstone quarrying in Jodhpur, this study may guide public health actions and policies to reduce mining-induced air pollution and related disorders.

## Data Availability

The datasets presented in this study can be found in online repositories. The names of the repository/repositories and accession number(s) can be found at: No.
