# Supplementary material for: Estimation of disease burden and multi-dimensional risk factors of chronic respiratory diseases among the residents near sandstone quarries in Jodhpur
Source: Front Public Health. 2026 Jan 12;13:1657608. doi: 10.3389/fpubh.2025.1657608 (PMC12833363; doi:10.3389/fpubh.2025.1657608)
Supplement: Supplementary file 1 [file Table_1.docx]

**Abbreviations**

ALRI - Acute Lower Respiratory Infections

AOR - Adjusted Odds Ratios

BPL - Below Poverty Line

CAT - COPD Assessment Test

CKD - Chronic Kidney Disease

CO - Carbon Monoxide

COPD - Chronic Obstructive Pulmonary Disease

CPCB - Central Pollution Control Board

CRD - Chronic Respiratory Disease

DM - Diabetes Mellitus

ENACs - Estimated numbers of attributable cases

GBD - Global Burden of Disease

GPS - Global Positioning System

HTN - Hypertension

JoDA - Jodhpur Development Authority

LPG - Liquefied Petroleum Gas

NAAQS - National Ambient Air Quality Standards

NAMP - National Air Quality Monitoring Programme

NCAP - National Clean Air Programme

NGO - Non-Governmental Organization

NO_2_ - Nitrogen Dioxide

PM - Particulate Matter

PNG - Piped Natural Gas

PMUY - Pradhan Mantri Ujjwala Yojana

P.T.B. - Pulmonary Tuberculosis

RSPCB - Rajasthan State Pollution Control Board

RSPM - Respiratory Suspended Particulate Matter

SPM - Suspended Particulate Matter

SO_2_ - Sulphur Dioxide

VOC - Volatile Organic Compounds

**Operational definitions**

**Chronic Respiratory Disease (CRD):** Chronic respiratory diseases (CRDs) negatively impact lung and airway function, and include conditions such as COPD, asthma, occupational lung diseases, and pulmonary hypertension. (World Health Organization, n.d.)

**COPD:** Chronic Obstructive Pulmonary Disease (COPD) is a diverse lung disorder marked by long-term respiratory symptoms such as shortness of breath, cough, sputum production, and frequent flare-ups. It results from abnormalities in the airways (such as bronchitis or bronchiolitis) and/or the alveoli (as seen in emphysema), leading to ongoing, typically worsening airflow limitation. (Kaminsky et al., 2022)

**Asthma:** The Global Initiative for Asthma (GINA) defines asthma as a diverse condition marked by persistent airway inflammation. It is characterized by a history of respiratory symptoms, including wheezing, shortness of breath, chest tightness, and coughing, which fluctuate in both intensity and frequency, along with varying limitations in expiratory airflow. (Halpin et al., 2021)

**Occupational lung diseases (OLD):** Occupational lung diseases (OLDs) are lung problems that are caused by occupational or workplace exposures. These conditions usually develop after long-term exposure to harmful substances in the air like dust, gases, vapors, or fumes at work place.( Minov, J. et al., 2022)

Occupational lung diseases caused by sclerogenic mineral dust (Silicosis, Anthrico-silicosis, Asbestosis) and silico-tuberculosis: provided that Silicosis is an essential factor in causing the resultant death.(Social Justice and Empowerment Department, 2019)

**Pneumonia:** According to Harrison’s textbook of internal medicine, pneumonia is an infection that affects the lung tissue and can be caused by different types of organisms. It’s not just single diseases, but a group of infections, each with its own causes, symptoms. (Levison, 2001)

**Pulmonary tuberculosis (PTB):** Pulmonary tuberculosis (PTB) refers to any bacteriologically confirmed or clinically diagnosed case of TB involving the lung parenchyma or the tracheobronchial tree.(World Health Organization, 2014)

**Non-Communicable Chronic diseases:** Noncommunicable diseases (NCDs), also known as chronic diseases, tend to be of long duration and are the result of a combination of genetic, physiological, environmental and behavioural factors. (World Health Organization, n.d.) These chronic conditions require long term or lifelong care, and include cancers, cardiovascular disease, stroke, chronic respiratory diseases, diabetes, mental health and neurological conditions, chronic kidney disease, among many others. (NCD Alliance, n.d.)

**Air Pollutants:** There are two main types of air pollutants: criteria pollutants and hazardous air pollutants (HAPs). Criteria pollutants include six common air pollutants particulate matter, ozone, sulfur dioxide, nitrogen dioxide, carbon monoxide, and lead. These are closely monitored because they pose a risk to public health and the environment. Hazardous air pollutants include 189 substances that are known to be toxic. These include chemicals like volatile organic compounds (VOCs), benzene, and formaldehyde, which can cause serious health problems such as cancer or damage to the nervous system.(Suh, Bahadori, Vallarino, & Spengler, 2000)

**Dust:** The World Health Organization (WHO) defined airborne dust as “solid particles, ranging in size from below 1 μm up to at least 100 μm, which may be or become airborne, depending on their origin, physical characteristics, and ambient conditions”.

**Daily smoker:** (smokes cigarettes every day) An adult who has smoked at least 100 cigarettes in his or her lifetime, and who now smokes every day. Previously called a “regular smoker”. (Centers for Disease Control and Prevention, n.d.)

**Occasional smoker:** (occasionally, or less than one cigarette per day) An adult who has smoked at least 100 cigarettes in his or her lifetime, who smokes now, but does not smoke every day. Previously called an “occasional smoker”. (Centers for Disease Control and Prevention, n.d.)

**Ex-smoker:** Person who had quit smoking at the time of interview. An adult who has smoked at least 100 cigarettes in his or her lifetime but who had quit smoking at the time of interview. (Centers for Disease Control and Prevention, n.d.)

**Never smoked:** An adult who has never smoked, or who has smoked less than 100 cigarettes in his or her lifetime. (Centers for Disease Control and Prevention, n.d.)

Current mine workers are sandstone mine workers who have at least 3 years of work experience.

Reference:

1. World Health Organization. (n.d.). *Chronic respiratory diseases*. World Health Organization.
2. Kaminsky, D. A., Mahler, D. A., Bhatt, S. P., Petty, T. L., Make, B. J., & Martinez, F. J. (2022). *Comprehensive care of patients with chronic obstructive pulmonary disease: Executive summary of the ATS/ERS clinical practice guideline*. American Journal of Respiratory and Critical Care Medicine, 206(11), 1293–1301. <https://doi.org/10.1164/rccm.202204-0671PP>
3. Halpin, D. M. G., Criner, G. J., Papi, A., Singh, D., Anzueto, A., Martinez, F. J., Agustí, A., & Celli, B. R. (2021). *Global initiative for the diagnosis, management, and prevention of chronic obstructive lung disease. The 2021 GOLD science committee report*. European Respiratory Journal, 59(1), 2102705. <https://pubmed.ncbi.nlm.nih.gov/34755626/>
4. Minov, J. *Occupational chronic obstructive pulmonary disorder: Prevalence and prevention. Expert Rev Respir Med. 2022; 16 (4): 429-36*.
5. Social Justice and Empowerment Department, Government of Rajasthan. (2019). *[Title of the PDF]*. <https://sje.rajasthan.gov.in/siteadmin/Uploads/201911211545271328.pdf>
6. ME, L. (1999). Pneumonia, including necrotizing pulmonary infections (lung abscess). *Harrison's principles of internal medicine*, 1437-1445.
7. World Health Organization. (2014). *Definitions and reporting framework for tuberculosis – 2013 revision*. World Health Organization. <https://www.who.int/tb/publications/definitions/en/>
8. World Health Organization. (n.d.). *Noncommunicable diseases*. World Health Organization. <https://www.who.int/news-room/fact-sheets/detail/noncommunicable-diseases>
9. NCD Alliance. (n.d.). *Noncommunicable diseases (NCDs)*. NCD Alliance. <https://ncdalliance.org/why-ncds/NCDs>
10. Suh, H. H., Bahadori, T., Vallarino, J., & Spengler, J. D. (2000). Criteria air pollutants and toxic air pollutants. *Environmental health perspectives*, *108*(suppl 4), 625-633.
11. WHO (1999) World Heath Organization Hazard prevention and control in the work environment: airborne dust. https://www.who. int/occupational_health/publications/airdust/en/. Accessed 6 Jun 2019
12. Centers for Disease Control and Prevention. (n.d.). *Tobacco glossary*. U.S. Department of Health & Human Services. <https://archive.cdc.gov/www_cdc_gov/nchs/nhis/tobacco/tobacco_glossary.htm>
